# Optimized UV-Spectrophotometric Assay to Screen Bacterial Uricase Activity Using Whole Cell Suspension

**DOI:** 10.3389/fmicb.2022.853735

**Published:** 2022-04-13

**Authors:** Benoit Pugin, Serafina Plüss, Denisa Mujezinovic, Rikke C. Nielsen, Christophe Lacroix

**Affiliations:** ^1^Laboratory of Food Biotechnology, Department of Health Sciences and Technology, ETH Zürich, Zürich, Switzerland; ^2^Beo Therapeutics AG, Zürich, Switzerland

**Keywords:** uricase, spectrophotometry, fluorescence, high-throughput screening, lactobacilli, *Bacillus*, *Bifidobacterium*

## Abstract

Uricase catalyzes the conversion of uric acid into allantoin with concomitant reduction of molecular oxygen to hydrogen peroxide. In humans, uricase is not functional, thereby predisposing individuals to hyperuricemia, a metabolic disturbance associated with gout, chronic kidney disorders, and cardiovascular diseases. The efficacy of current therapies to treat hyperuricemia is limited, and novel approaches are therefore desired, for instance using uricase-expressing probiotic strains. Here, we evaluated UV-spectrophotometric and H_2_O_2_-based fluorescent assays to enable the rapid identification of uricase activity in a broad panel of lactobacilli, *Bacillus*, and *Bifidobacterium* species. We highlighted abiotic (medium composition and mode of sterilization) and biotic (H_2_O_2_-producing strains) factors impacting the measurements’ accuracy, and reported on the stepwise optimization of a simple, fast, and robust high-throughput UV-spectrophotometric method to screen uricase activity using whole bacterial suspension, thereby assessing both cell-associated and extracellular activity. The validity of the optimized assay, based on the monitoring of uric acid degradation at 300 nm, was confirmed *via* liquid chromatography. Finally, a panel of 319 Qualified Presumption of Safety (QPS) strains of lactobacilli (18 species covering nine genera), *Bacillus* (three species), and *Bifidobacterium* (four species) were screened for uricase activity using the optimized method. All 319 strains, but the positive control *Bacillus* sp. DSM 1306, were uricase-negative, indicating that this activity is rare among these genera, especially in isolates from food or feces. Altogether, the UV-spectrophotometric high-throughput assay based on whole bacterial suspension reported here can be used to rapidly screen large microbial collections, by simultaneously detecting cell-associated and extracellular uricase activity, thereby accelerating the identification of uricolytic strains with therapeutic potential to treat hyperuricemia.

## Introduction

Uricase (urate oxidase; EC 1.7.3.3) is an enzyme involved in purine metabolism, which catalyzes the conversion of uric acid (UA) into allantoin with concomitant reduction of molecular oxygen to hydrogen peroxide (Kahn and Tipton, 1998). It is expressed in a wide range of organisms, including bacteria, fungi, plants, and animals (Kahn and Tipton, 1998). In humans, uricase is not functional due to multiple evolutionary events, resulting in 3- to 10-fold higher serum UA levels compared to other mammals (Kratzer et al., 2014) and predisposing humans to hyperuricemia. Hyperuricemia is a metabolic disturbance affecting ~20% of the United States population (Zhu et al., 2011) and causes gout, chronic kidney disorders, and cardiovascular diseases (Wu et al., 1994; Culleton et al., 1999). Hyperuricemia is exacerbated by western diet (Siener and Hesse, 2003) or other diets rich in purines (Brulé et al., 1992), thus therapeutic strategies often include nutritional and lifestyle interventions though evidence for their efficacy is scarce (Moi et al., 2013). On the other hand, UA-lowering drugs (e.g., xanthine oxidase inhibitors such as allopurinol and febuxostat) are restricted to patients with severe or recurrent gout (Engel et al., 2017), as they exhibit potential adverse effects (Pacher et al., 2006) and limited long-term efficacy (Pérez-Ruiz et al., 2019). Therefore, novel approaches are desired.

The gastrointestinal tract plays an important role in UA metabolism and excretion (Méndez-Salazar and Martínez-Nava, 2021). In healthy people, UA is primarily cleared *via* the kidneys, though ~30% is excreted *via* the intestine (Sorensen and Levinson, 1975) where it can be further degraded by the gut microbiota (Chu et al., 2021). In line, recent studies have shown that the gut microbial community composition is significantly altered in rat models of hyperuricemia (Liu et al., 2020; Pan et al., 2020) or in patients with gout (Chu et al., 2021; Lin et al., 2021). The gastrointestinal tract may therefore constitute a promising therapeutic target for hyperuricemia, for instance using oral probiotic strains, a concept supported by recent *in vivo* studies with microbial uricase (Szczurek et al., 2017) or uricolytic bacterial strains (García-Arroyo et al., 2018; Wu et al., 2021) that showed reduced serum UA levels in hyperuricemic rodent models.

To date, uricase activity was biochemically confirmed in various bacterial taxa, either extracellularly in *Pseudomonas* strains (Abdel-Fattah et al., 2005; Jagadeesan et al., 2019), or intracellularly in *Arthrobacter* (Suzuki et al., 2004), *Microbacterium* (Zhou et al., 2005; Kai et al., 2008), *Streptomyces* (Watanabe et al., 1969), *Saccharopolyspora* (Khucharoenphaisan and Sinma, 2011), *Micrococcus* (Olivieri et al., 1983), *Metabacillus* (Zhang et al., 2010), and *Bacillus* (Mahler, 1970; Bongaerts and Vogels, 1976; Bongaerts et al., 1978; Huang and Wu, 2004; Lee et al., 2005; Lotfy, 2008) strains. In few lactobacilli strains, uricase activity was suggested to be present both intracellularly and extracellularly (Handayani et al., 2018), thus prompting further investigation of uricase in taxa associated with probiotic strains. The determination of bacterial uricase activity can be assessed either qualitatively, *via* the observation of UA consumption on UA-containing solid media (Shaaban et al., 2015; Pustake et al., 2019), or quantitatively, *via* the measurement of H_2_O_2_ formation using fluorescent dyes (Fraisse et al., 2002) or the determination of UA degradation using spectrophotometry (Koyama et al., 1996; Fraisse et al., 2002; Huang and Wu, 2004) or liquid chromatography (Safranow et al., 2000). Because of their simplicity and compatibility with microplate systems allowing parallel analysis of multiple samples, spectrophotometric and fluorescent techniques appear promising to develop a high-throughput screening assay for bacterial uricase activity. However, their non-specific nature may be source of analytical artefacts, especially in presence of bacterial cells or complex media such as those used for the growth of probiotic strains.

Here, we evaluated both spectrophotometric and fluorescent uricase activity assays and highlighted abiotic and biotic factors potentially impacting the measurements’ accuracy and bias. We reported on the stepwise optimization of a simple, fast, and robust high-throughput UV-spectrophotometric method to screen bacterial uricase activity using whole bacterial suspension, thereby assessing both cell-associated and extracellular uricase. Finally, a panel of 319 QPS (Qualified Presumption of Safety) strains of lactobacilli (nine genera), *Bacillus*, and *Bifidobacterium*, respectively representing 18, 3, and 4 species, were screened for uricase activity using the optimized UV-spectrophotometric method.

## Materials and Methods

### Culture Media, Bacterial Strains, and Growth Conditions

The influence of eight different media on uricase activity measurement was evaluated, including: LactoBacillus Selection (LBS) and two different de Man, Rogosa, and Sharpe (MRS#1; NutriSelect™, Merck, Darmstadt, Germany. MRS#2; Biolife Italiana Srl, Milan, Italy) media for lactobacilli; Luria–Bertani (LB; Sigma-Aldrich, St. Louis, United States) and Allantoin Mineral (AM) media for *Bacillus* species; and Bifidus Selective Medium (BSM; NutriSelect™, Merck), Reinforced Clostridial Medium (RCM), and Wilkins-Chalgren (WC; Oxoid™, Thermo Scientific, Waltham, United States) media for *Bifidobacterium* species. Additionally, 2-fold diluted (½) MRS#1, MRS#2 and WC were evaluated. The detailed composition of all media is presented in Supplementary File 1. Media were either autoclaved (-Aut; 120°C, 20 min) or sterile filtered (-SF; Nalgene™ filter unit, 0.2 μm polyethersulfone membrane; Thermo Scientific). All media were freshly prepared the day before spectrophotometric and fluorescent measurements and stored at 4°C until analysis.

The 319 QPS strains used in this study (Supplementary Table S1) were obtained from our own collection, and consisted of 166 lactobacilli strains [18 species from nine genera; all formerly *Lactobacillus* (Zheng et al., 2020)], 110 *Bacillus* strains (three species), and 43 *Bifidobacterium* strains (four species) previously isolated from feces (human, mouse, and chicken) or fermented food products. *Bacillus* sp. DSM 1306 was acquired from the German Collection of Microorganisms and Cell Culture GmbH (DSMZ, Braunschweig, Germany). All strains were stored at −80°C in 25% (v/v) glycerol stocks and routinely grown at 37°C in 96-deep-well plates (Nolato Treff AG, Degersheim, Switzerland). *Bacillus* strains were grown aerobically in AM-Aut. Lactobacilli and *Bifidobacterium* strains were grown anaerobically using Oxoid™ AnaeroGen™ pouches (Thermo Scientific) in ½MRS#1-SF and ½WC-SF media, respectively. Prior to spectrophotometric and fluorescent analyses, the strains were reactivated in the corresponding media for 24 h, and pre-cultures were then transferred (1%, v/v) in fresh media and grown for 24 h before harvesting. When required, the medium was supplemented with 0.5 mM (fully soluble) or 30 mM (insoluble) uric acid.

### UV-Spectrophotometric Determination of Uricase Activity

Absorbance spectra of the tested growth media, measured in absorbance units (AU), were determined between 250 and 350 nm in UV-transparent (acrylic; Corning Inc., New York, United States) or standard (polystyrene; SPL Life Sciences Co. Ltd., Gyeonggi-do, South Korea) 96-well plates by mixing 40 μl medium with 160 μl borate buffer (5 mM H_3_BO_3_, pH 8.5).

Spectrophotometric uricase activity was determined by monitoring the reduction of uric acid at 300 nm (AU_300_), 37°C, aerobically, for 60 min in standard 96-well plate. The assay was initiated by mixing 40 μl sample or uricase (70 mU/ml final concentration; product No. U0880, Sigma-Aldrich) with 160 μl UA (0.5 mM final) in borate buffer. The samples tested included fresh medium, whole culture samples (cells and supernatant), supernatants (5,500 × *g*, 10 min, 4°C), or the cell pellet washed once and resuspended in PBS (pH 7.4). Uricase activity was calculated using the linear region of the spectrophotometric kinetic curve corresponding to the maximum reaction rate. Uric acid concentration was determined from absorbance readings using UA standard curves generated in the 0.1–0.5 mM range. One unit of uricase is defined as the amount of sample that consumes 1.0 μmol of UA per minute under the standard assay conditions.

All spectrophotometric analyses were performed with a preheated microplate spectrophotometer PowerWave™ XS (BioTek instruments, Winooski, United States).

### Fluorescent Determination of Uricase Activity

Uricase activity was determined using the fluorescent Amplex™ Red Uric Acid/Uricase Assay Kit (Thermo Scientific) to quantify H_2_O_2_ production and was expressed as relative fluorescence units (RFU). Kinetics were performed at 37°C, aerobically, for 60 min in black 96-well plate with transparent bottom (polystyrene; Greiner Bio-One GmbH, Frickenhausen, Germany) in a preheated microplate fluorescent reader FL600 (BioTek instruments) with excitation and emission bandpass filters of 485/20 and 635/20 nm, respectively. All solutions were provided in the kit. Briefly, 20 μl sample (medium or bacterial suspension) or uricase (5 mU/ml final) was mixed with 69.3 μl reaction buffer, 10 μl UA (0.5 mM final), 0.2 μl horseradish peroxidase (0.2 U/ml final), and 0.5 μl Amplex Red (50 μM final). Uricase activity was calculated using the linear region of the fluorescent kinetic curve corresponding to the maximum reaction rate. Hydrogen peroxide concentration was determined from fluorescent readings using H_2_O_2_ standard curves generated in the 0–0.01 mM range. One unit of uricase is defined as the amount of sample that produces 1.0 μmol of H_2_O_2_ per minute under the standard assay conditions.

### Quantification of Uric Acid by Liquid Chromatography

Uric acid was quantified by Ultra High Performance Liquid Chromatography equipped with a Diode Array Detector (UHPLC-DAD), modified from Safranow et al. (2000). Uric acid 10 mM standard stock solution (analytical grade; Sigma-Aldrich) was prepared in 25 mM NaOH and was further diluted with MilliQ water to generate a standard curve. Bacterial suspensions were centrifuged (14,000 × *g*, 10 min, 4°C), and supernatants were filtered (0.45 μm nylon membrane filter) prior to UHPLC-DAD analysis. The separation was carried out with a Vanquish™ Flex UHPLC System (Thermo Scientific), coupled to an ACQUITY BEH C18 column (1.7 μm particle size, 2.1 × 100 mm; Waters Corp., Milford, United States). Samples (1 μl injection) were eluted at 24°C with a 0.2 ml/min flow rate under isocratic conditions using 50 mM phosphate buffer pH 5.5 / methanol (97/3, *v/v*) as mobile phase. UA was quantified using a Vanquish™ diode array detector at 300 nm. Data were processed using Chromeleon 7 software (Thermo Scientific).

## Results

### Influence of Media Composition and Sterilization on Basal UV-Spectrophotometric Absorbance

**Figure 1:**
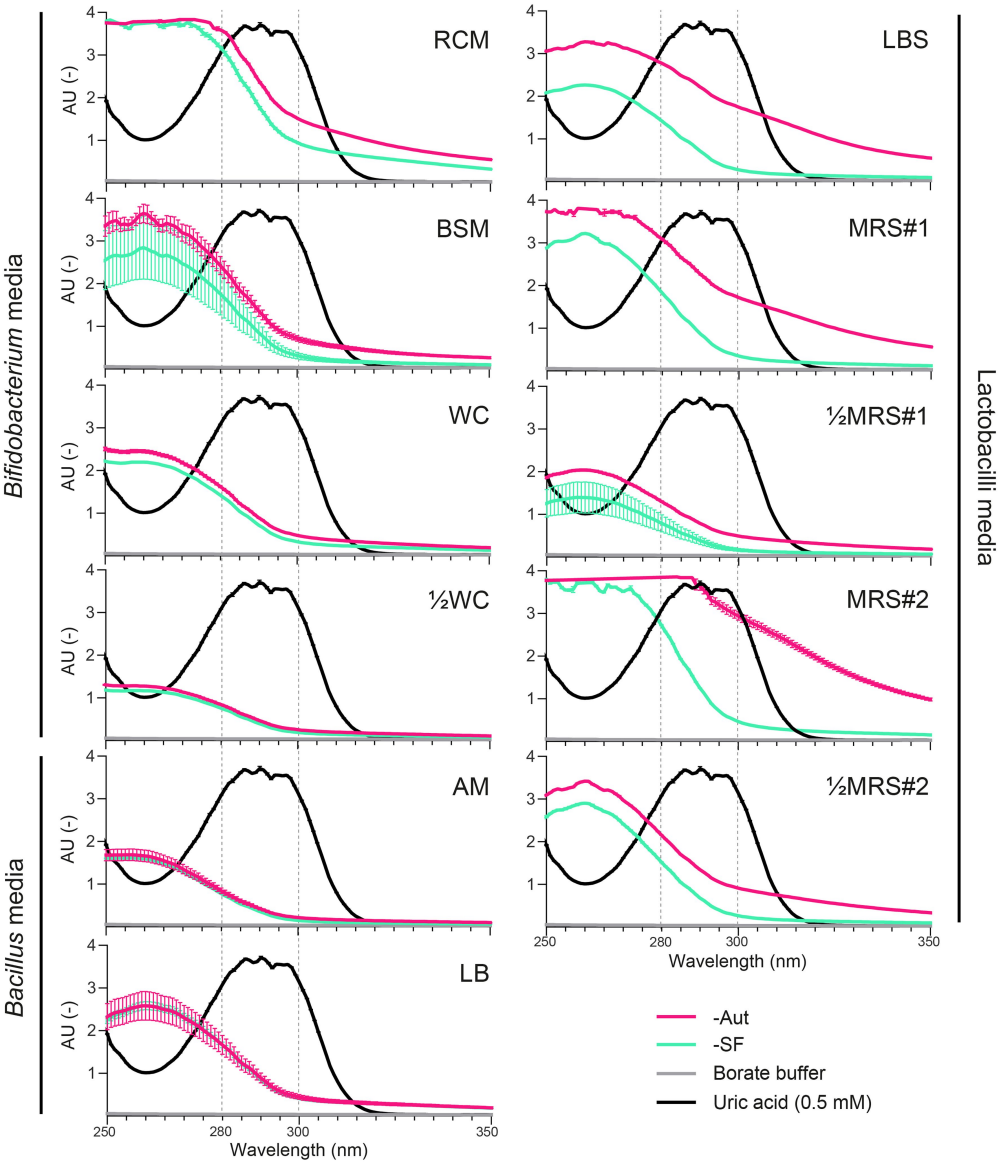
Absorbance spectra (250–350 nm; UV-transparent 96-well plate) of uric acid (0.5 mM in borate buffer) compared to autoclaved (-Aut) or sterile filtered (-SF) media commonly used for the growth of lactobacilli, *Bacillus*, and *Bifidobacterium* species. AU, absorbance units. Data represent the mean and SD from two independent replicates.

We first evaluated whether the spectrophotometric quantification of UA could be impaired by major UV-absorbing compounds present in common growth media or generated during sterilization, i.e., autoclaved (Aut) vs. sterile filtered (SF). Absorbance spectra in a UV-transparent 96-well plate showed maximal absorbance of UA in the 280–300 nm range (>3.00 AU), where all media also displayed basal levels of absorbance, which decreased as wavelengths increased (Figure 1). In the maximal absorbance range of UA, the autoclaved medium MRS#2-Aut absorbed most, at level similar or higher than pure UA (≥3.00 AU), whereas ½WC, AM (Aut and SF) and sterile filtered ½MRS#1-SF absorbed least (<1.00 AU; Figure 1). The mode of sterilization had an important impact on the absorbance of most media, including RCM, BSM, LBS, MRS#1, ½MRS#1, MRS#2, and ½MRS#2, but not WC, ½WC, AM, and LB. Overall, autoclaved media displayed higher absorbance levels than sterile filtered media, which was particularly evident for LBS-Aut, MRS#1-Aut, and MRS#2-Aut with an AU increase of up to 1.51 (292–293 nm), 1.40 (290–296 nm), and 2.50 (296–297 nm) compared to their SF counterpart (Figure 1). Using a standard polystyrene 96-well plate, similar absorbance patterns were observed across the tested conditions, except for the strong plate absorbance measured at low wavelengths (> 3.00 AU at ≤280 nm; Supplementary Figure S1) compared to UV-transparent 96-well plate. At 300 nm, the polystyrene plate background absorbance was moderate (0.40 AU), and this wavelength was thus selected for subsequent UV-spectrophotometric assessment of bacterial uricase activity with standard 96-well plate. It is worth noting that the UA precursor xanthine and the UA degradation product allantoin did not absorb at 300 nm (data not shown).

### Influence of Media Composition and Sterilization on Fluorescent Measurements

**Figure 2:**
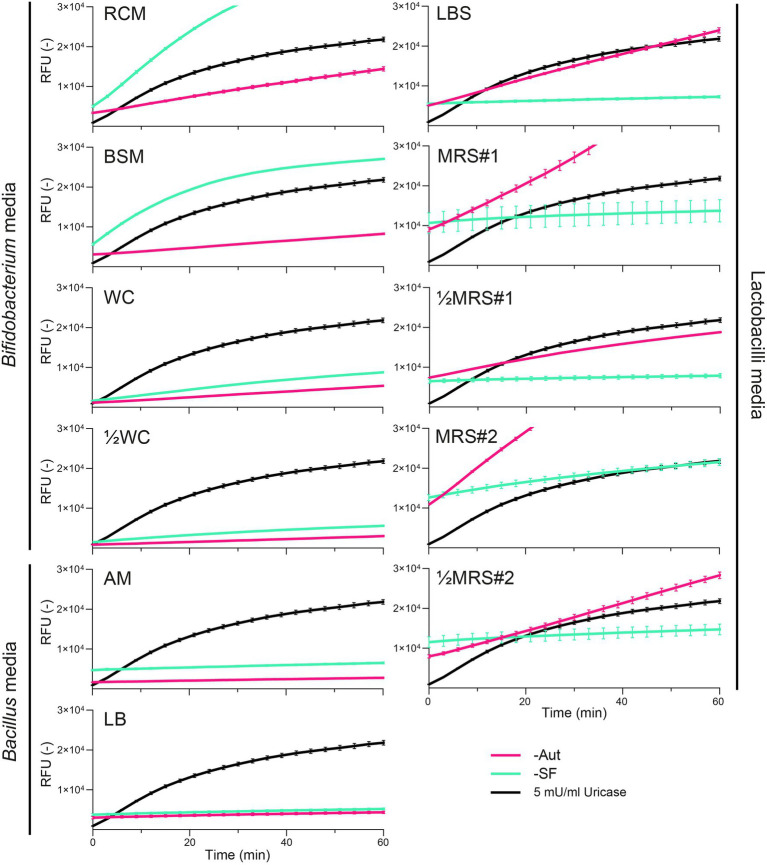
Fluorescence kinetics of uricase (5 mU/ml) compared to autoclaved (-Aut) or sterile filtered (-SF) complex media commonly used for the growth of lactobacilli, *Bacillus*, and *Bifidobacterium* species. RFU, relative fluorescence units. Data represent the mean and SD from two independent replicates.

We next evaluated whether growth media and their sterilization could also interfere with fluorescent assays based on the quantification of H_2_O_2_ as a marker byproduct of the uricase activity. All media displayed basal levels of fluorescence and surprisingly these intensities appeared to increase over time (Figure 2). The highest rates (ΔRFU/Δtime) were observed for MRS#2-Aut and RCM-SF, which would correspond to a uricase activity of 7.26 ± 0.18 and 7.41 ± 0.01 mU/ml, respectively (Supplementary Table S2). The mode of sterilization impacted the fluorescence measurements, with distinct intensities observed between Aut and SF conditions in RCM, BSM, LBS, MRS#1, ½MRS#1, MRS#2, ½MRS#2, and to a lesser extend in WC, ½WC, AM, and LB (Figure 2). However, autoclaved media did not always yield stronger fluorescence rates: LBS-Aut, MRS#1-Aut, ½MRS#1-Aut, MRS#2-Aut, and ½MRS#2-Aut showed higher fluorescence rates compared to their SF counterpart, whereas BSM-SF and RCM-SF showed higher fluorescence rates compared to their Aut counterpart (Figure 2).

### Evaluation of Uricase Activity Using Whole Bacterial Suspension

To minimize the impact of the nutritional medium on UV-spectrophotometric and fluorescent analyses, ½MRS#1-SF, AM-Aut, and ½WC-SF were selected for the growth and screening of lactobacilli, *Bacillus*, and *Bifidobacterium* species, respectively. UV-spectrophotometric and fluorescent methods were then evaluated in presence of whole bacterial culture samples to measure simultaneously both extracellular and cell-associated uricase activity, and identify potential cofounding factors. Five strains were tested including *Bacillus* sp. DSM 1306 as uricase activity positive control, and randomly selected strains, *Levilactobacillus brevis* BT-4087, *Lacticaseibacillus rhamnosus* BT-1025, *Bifidobacterium* sp. BT-4055X, and *Bifidobacterium bifidum* BT-4055Y.

**Figure 3:**
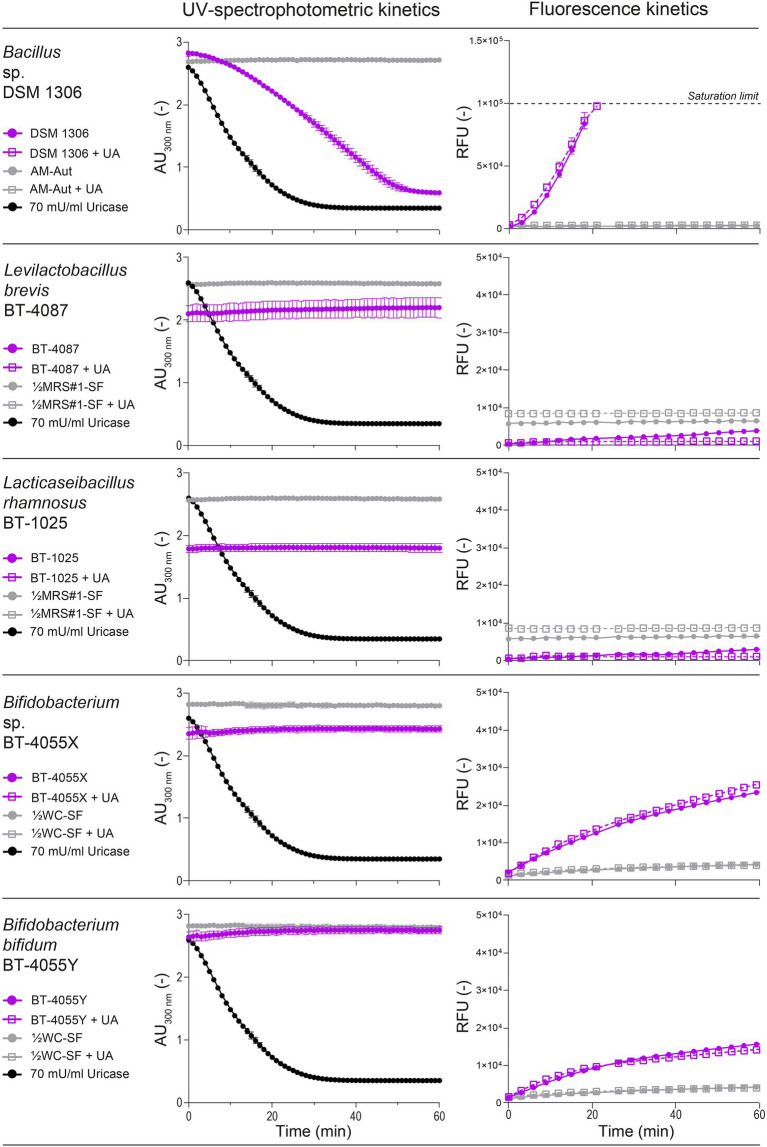
Evaluation of uricase activity using *Bacillus* (DSM 1306), lactobacilli (BT-4087 and BT-1025), and *Bifidobacterium* (BT-4055X and BT-4055Y) suspension (cells and supernatants) as determined by UV-spectrophotometric (absorbance at 300 nm) and fluorescence assays. Strains were grown in the corresponding medium for 24 h in presence or absence of 30 mM uric acid (UA). Data represent the mean and SD from two biological replicates.

When comparing both methods, the positive control *Bacillus* sp. DSM 1306 showed similar uricase activity *via* spectrophotometry (37.33 ± 2.17 mU/ml) and fluorescence (38.27 ± 1.79 mU/ml; Figure 3), and the activity appeared to be cell-associated and absent from the supernatant (Supplementary Figure S2). In contrast, lactobacilli and *Bifidobacterium* strains showed no reduction of UA at 300 nm, but both *Bifidobacterium* strains BT-4055X and BT-4055Y showed increased fluorescence intensity corresponding to 4.19 ± 0.03 and 3.25 ± 0.05 mU/ml uricase activity equivalences, respectively (Figure 3). To clarify the observed discrepancy between both assays and confirm the presence/absence of uricolytic activity in the tested strains, all five strains were grown in presence of 0.5 mM UA for 2 or 24 h prior to harvesting, and the remaining UA was quantified *via* UHPLC-DAD analysis. We confirmed that *Bacillus* sp. DSM 1306 could degrade most UA present in the medium (−0.44 ± 0.01 and −0.45 ± 0.00 mM after 2 and 24 h incubation, respectively), but no reduction of UA was observed with any lactobacilli or *Bifidobacterium* strains at both incubation times (Supplementary Table S3). This data suggests that the increased fluorescence observed with *Bifidobacterium* BT-4055X and BT-4055Y was unrelated to uricase activity. Finally, it is worth noting that the supplementation of 30 mM UA in the media during growth did not stimulate uricase activity in *Bacillus* DSM 1306 compared with the UA-free growth conditions, nor induce uricase activity in the tested lactobacilli and *Bifidobacterium* strains (Figure 3).

### Screening of Uricase Activity in Lactobacilli, *Bacillus*, and *Bifidobacterium* Species

**Figure 4:**
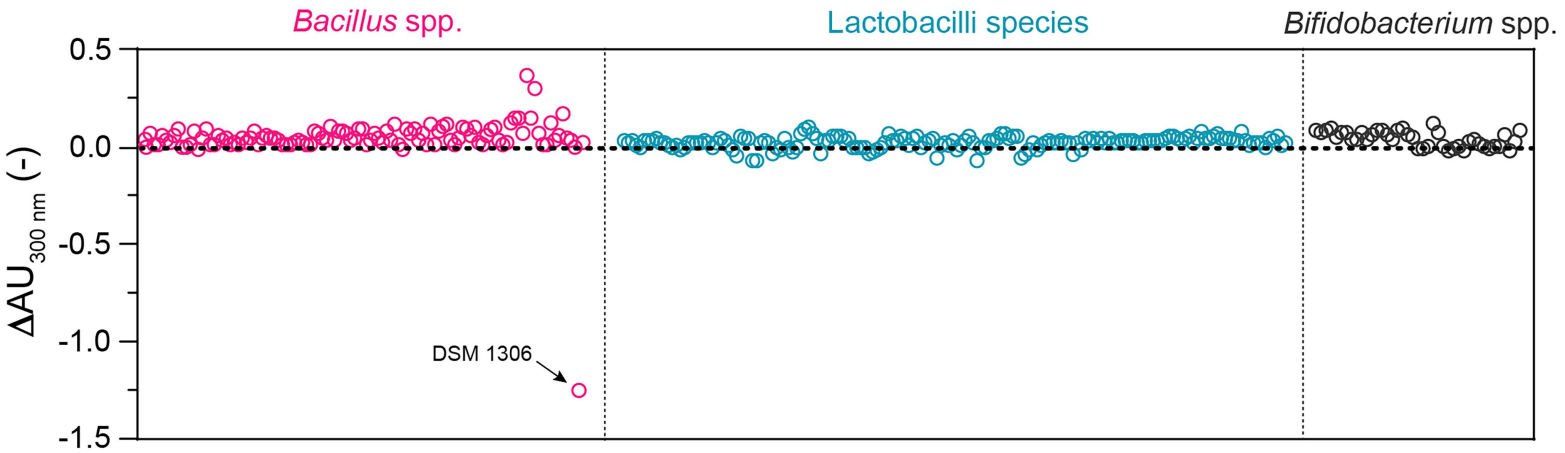
Uricase activity in a panel of 319 QPS strains of *Bacillus* (*n* = 110; three species), lactobacilli (*n* = 166; 18 species from nine genera), and *Bifidobacterium* (*n* = 43; four species) as determined by UV-spectrophotometry at 300 nm in standard 96-well plate. Each dot corresponds to the difference of absorbance (ΔAU_300_) over 60 min generated by the bacterial suspension of a specific strain (one biological replicate; all strains tested are listed in Supplementary Table S1). *Bacillus* sp. DSM 1306 was used as positive control.

Altogether, the UV-spectrophotometric assay emerged as the most simple, effective, and robust method for high-throughput screening of bacterial uricase activity, and was therefore used to screen a large panel of 319 QPS strains of lactobacilli (*n* = 166), *Bacillus* (*n* = 110), and *Bifidobacterium* (*n* = 43; Supplementary Table S1). Beside the positive control *Bacillus* sp. DSM 1306, none of the strains from these genera showed reduction of absorbance at 300 nm over 60 min, indicating the absence of uricase activity under the tested conditions (Figure 4).

## Discussion

In this work, we evaluated two quantitative methods to develop a high-throughput screening approach of bacterial uricase activity in microtiter plates, with particular focus on conditions relevant to lactobacilli, *Bacillus*, and *Bifidobacterium* strains. Using a stepwise approach, we aimed at developing a sensitive, yet robust method using whole bacterial suspensions to enable the simultaneous detection of both extracellular and cell-associated uricase activities, thereby accelerating the screening of large microbial collections and the identification of uricolytic strains independently from the enzyme’s partition (Watanabe et al., 1969; Olivieri et al., 1983; Suzuki et al., 2004; Kai et al., 2008; Lotfy, 2008; Zhang et al., 2010; Khucharoenphaisan and Sinma, 2011; Handayani et al., 2018; Jagadeesan et al., 2019).

Our data revealed that the fluorescent assay, based on the quantification of H_2_O_2_ released during UA degradation, was the least suitable method because of its susceptibility to biases derived from both abiotic and biotic factors. In absence of bacteria, many media exhibited strong background fluorescence intensities (=false positive), as exemplified by the fluorescence rates of MRS#2-Aut and RCM-SF which corresponded to ~20% of the fluorescence rate of the uricase positive control *Bacillus* sp. DSM 1306 (Supplementary Table S2, Figure 3). This abiotically-generated fluorescence might have arisen from the spontaneous generation of H_2_O_2_ as byproduct from the reactions between sugars, phosphate salts and proteinaceous components (e.g., tryptone, peptone, and yeast extract), especially during heat treatment (Finkelstein and Lankford, 1957; Carlsson et al., 1978; Nakashima et al., 2010). More importantly, the fluorescent assay was prone to false positive signals in presence of bacterial cells (Figure 3). As previous studies showed that H_2_O_2_ can be produced by various *Bifidobacterium* (Kawasaki et al., 2009) and lactobacilli (Pridmore et al., 2008; Martín and Suárez, 2010; Hertzberger et al., 2014) strains, the H_2_O_2_-based fluorescent assay appears unsuitable to screen uricase activity using whole bacterial suspensions.

In contrast, the UV-spectrophotometric method evaluated and optimized here can be used for the high-throughput screening of bacterial uricase activity. By monitoring UA degradation at 300 nm, we maximized the absorbance of UA and minimized the background absorbance of most tested complex media (Figure 1), while enabling the use of standard polystyrene 96-well plates (Supplementary Figure S1, Figure 4). To ensure the sensitivity of the spectrophotometric measurements, medium selection and preparation should be carefully considered though. Thermal sterilization by autoclaving consistently increased the background absorbance of most media (Figure 1), probably due to the reaction between reducing sugars (e.g., glucose) and proteinaceous components resulting in Maillard reaction products (Hemmler et al., 2017) that absorbed in the UV–Vis wavelengths range (Wang et al., 2016; Liu et al., 2018). The absence or low concentration of glucose in WC, ½WC, AM, and LB (Supplementary File 1) could explain the minor variations of absorbance observed between these media when autoclaved or filter sterilized (Figure 1). It is worth noting that autoclaved MRS, a media heavily used in laboratories to grow lactobacilli strains, were highly absorbing at UV wavelengths, thus hindering its compatibility with UV-spectrophotometric assays in general (Figure 1); nonetheless, those effects can be attenuated by using a diluted and sterile filtered version of this medium (Supplementary File 1).

The screening of 319 QPS strains of lactobacilli (nine genera), *Bacillus*, and *Bifidobacterium* using the optimized UV-spectrophotometric assay did not result in the identification of uricase positive strains, except for the positive control *Bacillus* sp. DSM 1306. Although we cannot fully discard the possibility of few false negative results from the high-throughput screening, previous reports on the regulation of bacterial uricase activity support the conditions tested here. Nitrogen- or carbon-limitation was shown to stimulate uricase activity in resting cells of *Streptomyces* spp. (Watanabe et al., 1976), thereby validating the use of protein-depleted media to grow and assess bacterial uricase activity (i.e., ½MRS#1-SF, AM-Aut, and ½WC-SF; Supplementary File 1). In various *Bacillus* species, UA was shown to serve as a nitrogen source (Bongaerts and Vogels, 1976), and while no UA was supplemented in the culture media of the 319 tested strains, cells were grown in protein-depleted media, harvested in the stationary growth phase, and ultimately exposed to 0.5 mM UA for 60 min during uricase activity measurement (Figure 4). Besides, uricase activity was not promoted by the direct supplementation of UA in the growth media of four randomly tested strains, and the positive control *Bacillus* sp. DSM 1306 (Figure 3).

The data obtained from the 319 screened strains, representing a total of 11 genera and 25 species with several strains of the same species (Supplementary Table S1), strongly suggest that uricase activity is a rare feature in lactobacilli, *Bacillus*, and *Bifidobacterium* species, especially in strains isolated from fermented food products or from the gastrointestinal tract. Beside previous reports on uricase-positive *Bacillus* strains from soil samples (Mahler, 1970; Bongaerts and Vogels, 1976; Huang and Wu, 2004; Lotfy, 2008), no study have yet reported the presence of uricase in *Bifidobacterium*, and only two studies reported uricase activity in lactobacilli food isolates, i.e., *Limosilactobacillus fermentum* (Wu et al., 2021) and *Lactiplantibacillus plantarum* (Handayani et al., 2018). Considering that 40 *L. fermentum* and 29 *L. plantarum* strains (Supplementary Table S1) were tested uricase-negative in our screening, and that no uricase-encoding gene has yet been identified in lactobacilli genomes (in contrast to the uricase-encoding genes in *Bacillus*; Schultz et al., 2001), we question whether previous reports on lactobacilli uricase activity (Handayani et al., 2018; Wu et al., 2021) are the results of potential methodological biases as highlighted here for a broad panel of tested strains from 11 genera, or a less likely highly strain-specific feature.

Altogether, the whole bacterial suspension screening method developed here provides various advantages compared to the qualitative agar plate-based assay, as it is quantitative, specific, and scalable. Our approach can serve as a guide for the assessment of uricase activity in large and taxonomically diverse microbial collection, and therefore potentially supporting the identification of novel uricolytic strains with therapeutic potential for the treatment of hyperuricemia and associated comorbidities.

## Data Availability Statement

The original contributions presented in the study are included in the article/**Supplementary Material**; further inquiries can be directed to the corresponding author.

## Author Contributions

BP, SP, DM, and CL designed the experiments. RN provided critical feedback on the study design. SP and DM performed the experiments. BP, SP, and DM performed data analysis. BP and CL wrote the manuscript. All authors contributed to the article and approved the submitted version.

## Funding

This work was supported by a grant from the Swiss Innovation Agency (40722.1 IP-LS), in collaboration with Beo Therapeutics AG. The research was conducted in the absence of any commercial relationships that could have influenced or biased the work presented here.

## Conflict of Interest

RN works for the company Beo Therapeutics AG.

The remaining authors declare that the research was conducted in the absence of any commercial or financial relationships that could be construed as a potential conflict of interest.

## Publisher’s Note

All claims expressed in this article are solely those of the authors and do not necessarily represent those of their affiliated organizations, or those of the publisher, the editors and the reviewers. Any product that may be evaluated in this article, or claim that may be made by its manufacturer, is not guaranteed or endorsed by the publisher.
