# Supplementary material for: Optimized UV-Spectrophotometric Assay to Screen Bacterial Uricase Activity Using Whole Cell Suspension
Source: Front Microbiol. 2022 Apr 13;13:853735. doi: 10.3389/fmicb.2022.853735 (PMC9043897; doi:10.3389/fmicb.2022.853735)
Supplement: Supplementary file 1 [file Data_Sheet_1.zip › Supplementary Tables_Figures.docx]

**Supplementary tables and figures:**

**Table S1: Bacterial strains used in this study.**

| **Species** | **Number of strains** |
| --- | --- |
| *Bacillus* sp. DSM 1306 | 1 |
| *Bacillus amyloliquefaciens* | 46 |
| *Bacillus subtilis* | 62 |
| *Bacillus vallismortis* | 2 |
| *Bifidobacterium* sp. | 1 |
| *Bifidobacterium bifidum* | 15 |
| *Bifidobacterium breve* | 17 |
| *Bifidobacterium longum* | 10 |
| *Lacticaseibacillus casei* | 16 |
| *Lacticaseibacillus paracasei* | 7 |
| *Lacticaseibacillus rhamnosus* | 9 |
| *Lactiplantibacillus plantarum* | 29 |
| *Lactobacillus acidophilus* | 2 |
| *Lactobacillus delbrueckii* | 23 |
| *Lactobacillus helveticus* | 3 |
| *Lactobacillus johnsonii* | 1 |
| *Latilactobacillus curvatus* | 11 |
| *Latilactobacillus sakei* | 1 |
| *Lentilactobacillus buchneri* | 5 |
| *Levilactobacillus brevis* | 13 |
| *Ligilactobacillus salivarius* | 1 |
| *Limosilactobacillus fermentum* | 40 |
| *Limosilactobacillus mucosae* | 1 |
| *Limosilactobacillus pontis* | 1 |
| *Limosilactobacillus reuteri* | 2 |
| *Loigolactobacillus coryniformis* | 1 |

**Table S2:** **Uricase activity (mU/ml; false positive) calculated from the background fluorescence generated by autoclaved (Aut) and sterile filtered (SF) media.** Data represent the mean and standard deviation from two independent replicates.

| **Medium** | **Uricase activity (mU/ml)** | |
| --- | --- | --- |
|  | **Aut** | **SF** |
| **RCM** | 1.59 ± 0.07 | 7.41 ± 0.01 |
| **BSM** | 0.65 ± 0.03 | 5.32 ± 0.09 |
| **WC** | 0.53 ± 0.00 | 1.10 ± 0.01 |
| **½WC** | 0.27 ± 0.00 | 0.70 ± 0.00 |
| **AM** | 0.15 ± 0.00 | 0.27 ± 0.03 |
| **LB** | 0.22 ± 0.00 | 0.23 ± 0.00 |
| **LBS** | 2.67 ± 0.10 | 0.28 ± 0.00 |
| **MRS#1** | 4.61 ± 0.15 | 0.59 ± 0.10 |
| **½MRS#1** | 1.86 ± 0.00 | 0.25 ± 0.00 |
| **MRS#2** | 7.26 ± 0.18 | 1.53 ± 0.03 |
| **½MRS#2** | 2.53 ± 0.07 | 0.55 ±0.03 |

**Table S3: Change in UA concentration (mM; determined by UHPLC-DAD) in bacterial cultures incubated with 0.5 mM uric acid for 2 h or 24 h prior to analysis**. *Bacillus* sp. DSM 1306 was grown aerobically at 37°C in AM-Aut. Lactobacilli and *Bifidobacterium* strains were grown anaerobically at 37°C in ½MRS#1-SF and ½WC-SF, respectively. Data represent the mean and standard deviation from two independent biological replicates.

| **Strain** | **Δ Uric acid (mM)** | |
| --- | --- | --- |
|  | 2 h exposure | 24 h exposure |
| *Bacillus* sp. DSM 1306 | -0.44 ± 0.01 | -0.45 ± 0.00 |
| *Levilactobacillus brevis* BT-4087 | -0.03 ± 0.01 | -0.05 ± 0.02 |
| *Lacticaseibacillus rhamnosus* BT-1025 | 0.00 ± 0.05 | 0.01 ± 0.01 |
| *Bifidobacterium* sp. BT-4055X | -0.03 ± 0.01 | 0.02 ± 0.01 |
| *Bifidobacterium* *bifidum* BT-4055Y | 0.01 ± 0.01 | 0.02 ± 0.03 |


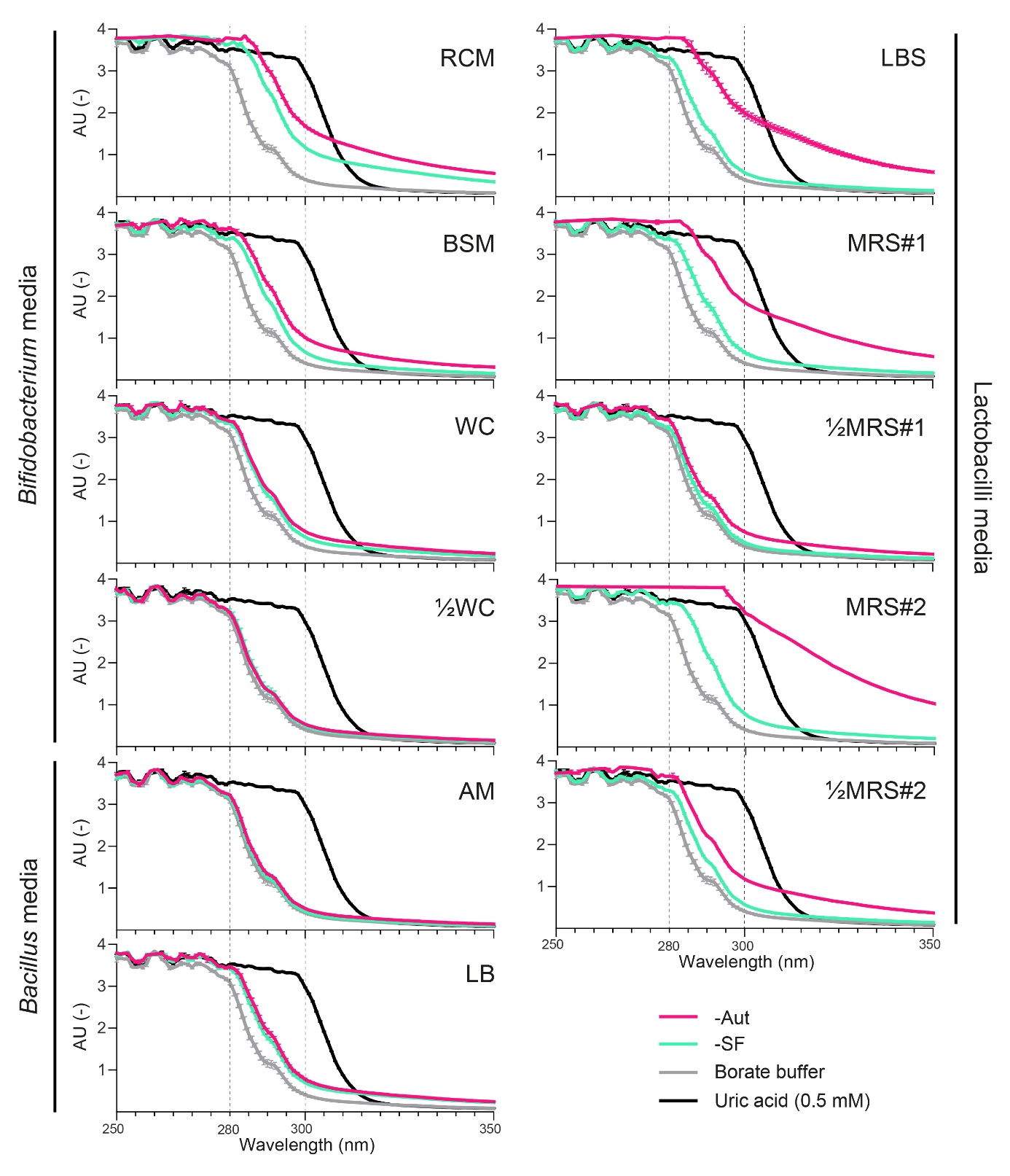


**Figure S1: Absorbance spectra (250-350 nm; standard 96-well plate) of uric acid (0.5 mM in borate buffer) compared to autoclaved (-Aut) or sterile filtered (-SF)** **media commonly used for the growth of lactobacilli, *Bacillus* and *Bifidobacterium* species.** AU: Absorbance Units. Data represent the mean and standard deviation from two independent replicates.

**Figure S2: Evaluation of uricase activity of *Bacillus* sp. DSM 1306** using whole bacterial suspension (cells and supernatant; n = 2), supernatant (n = 5), and cell pellet (washed and resuspended in PBS; n =1). AM-Aut medium (n = 3) was used as negative control. Data represent the mean and standard deviation.
